# *Arabidopsis* peroxisome proteomics

**DOI:** 10.3389/fpls.2013.00101

**Published:** 2013-04-24

**Authors:** John D. Bussell, Christof Behrens, Wiebke Ecke, Holger Eubel

**Affiliations:** ^1^Australian Research Council Centre of Excellence in Plant Energy Biology, The University of Western AustraliaCrawley, WA, Australia; ^2^Institute for Plant Genetics, Leibniz Universität HannoverHannover, Germany

**Keywords:** peroxisome, subcellular localization, protein:protein interaction, free-flow electrophoresis, functional proteomics, targeted quantitation of proteins

## Abstract

The analytical depth of investigation of the peroxisomal proteome of the model plant *Arabidopsis thaliana* has not yet reached that of other major cellular organelles such as chloroplasts or mitochondria. This is primarily due to the difficulties associated with isolating and obtaining purified samples of peroxisomes from *Arabidopsis*. So far only a handful of research groups have been successful in obtaining such fractions. To make things worse, enriched peroxisome fractions frequently suffer from significant organellar contamination, lowering confidence in localization assignment of the identified proteins. As with other cellular compartments, identification of peroxisomal proteins forms the basis for investigations of the dynamics of the peroxisomal proteome. It is therefore not surprising that, in terms of functional analyses by proteomic means, peroxisomes are lagging considerably behind chloroplasts or mitochondria. Alternative strategies are needed to overcome the obstacle of hard-to-obtain organellar fractions. This will help to close the knowledge gap between peroxisomes and other organelles and provide a full picture of the physiological pathways shared between organelles. In this review, we briefly summarize the status quo and discuss some of the methodological alternatives to classic organelle proteomic approaches.

## INTRODUCTION

**FIGURE 1:**
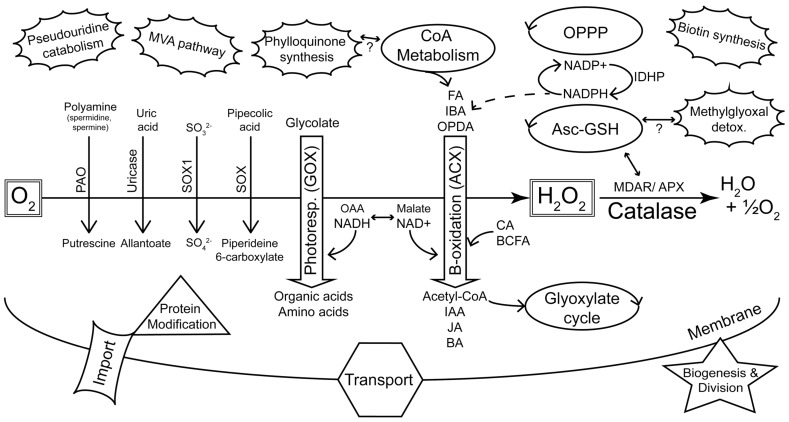
**Summary of major metabolic pathways and processes of plant peroxisomes as identified by proteomics.** The peroxisome plays a key role in sequestering reactions that evolve reactive oxygen species (de Duve, 1969a). This is highlighted by the diverse oxidases (downward pointing arrows) that generate H_2_O_2_, and the detoxification of ROS by catalase and other enzymes. Photorespiration and β-oxidation are emphasized in the center of the figure as the major pathways. The dehydrogenases of β-oxidation are primarily dependent on NAD^+^ as an electron acceptor. Hydroxypyruvate reductase (HPR) of photorespiration requires NADH and this shift in redox requirements is depicted by the interchange between OAA/NADH on the one hand and malate/NAD^+^ on the other. NADPH is also required by β-oxidation for pathways including unsaturated FA catabolism and JA synthesis. Peroxisome-localized pathways (e.g., glyoxylate cycle, CoA metabolism, OPPP, Asc-GSH cycle) that can be linked with classic peroxisome metabolism are indicated as regular ellipses. The unconnected (pseudouridine catabolism, MVA pathway, biotin synthesis) or tentatively connected (phylloquinone synthesis, methylglyoxal detoxification) clouds are new additions to the list of known or proposed peroxisome-localized pathways (Reumann, 2011) and are not readily related to core peroxisome metabolism. *KEY*: *Oxidases*: PAO, polyamine oxidase; SOX, sarcosine oxidase; SOX1, sulfite oxidase; GOX, glycolate oxidase; ACX, acyl-CoA oxidase. *Substrate classes*: FA, fatty acid; IBA, indole-3-butyric acid; OPDA, 12-oxo-phytodienoic acid; CA, cinnamic acid; BCFA, branched chain fatty acid; *Product classes*: IAA, indole-3-acetic acid; JA, jasmonic acid; BA, benzoic acid; OAA, oxaloacetic acid. *Other enzymes and pathways*: MDAR, monodehydroascorbate reductase; APX, ascorbate peroxidase; Asc-GSH, ascorbate-glutathione cycle; OPPP, oxidative pentose phosphate pathway; MVA, mevalonate; IDHP, NADP-dependent isocitrate dehydrogenase.

Microbodies were discovered in the mid 1950s as a particular structure visible in electron micrographs of mouse kidney and rat liver cells (de Duve and Baudhin, 1966). The organelles were initially characterized by co-precipitating enzymatic activities and were consequently named peroxisomes due to the co-precipitation of oxidases and hydrogen peroxide metabolism (catalase) with the isolated structures (de Duve and Baudhin, 1966). Peroxisomes were discovered in plants in the late 1960s due to their association with enzymes of photorespiration (Tolbert et al., 1968, 1969) and subsequently found in most eukaryotic organisms (de Duve, 1969a). These early studies showed that peroxisomes primarily housed reactions that yielded reactive oxygen species (ROS; including oxidase reactions of β-oxidation and purine metabolism) and enzymes (e.g., catalase) to detoxify ROS. In addition, enzymes of the photorespiratory pathway were found in plant peroxisomes (**Figure 1**).

Up until about 10 years ago understanding of the basic function of peroxisomes in plants had not changed significantly from those early discoveries. Primarily, enzyme activities that completed already known peroxisomal pathways were identified. These included, for example, peroxisomal thiolase in the β-oxidation pathway of plants (Cooper and Beevers, 1969) which previously had only acyl-CoA oxidase (ACX), β-hydroxyacyl-CoA dehydrogenase (multi-functional protein, MFP) and enoyl-CoA hydratase (MFP) activities defined (de Duve, 1969a). More recently, the availability of the genomic sequence of *Arabidopsis thaliana* (Arabidopsis Genome Initiative, 2000) provided a significant boost to investigations of peroxisome function. For the first time, a whole genome could be interrogated using algorithms designed for prediction of protein subcellular localization signals (as discussed below). Moreover, comparative genomic tools have meant that homologs from unrelated species could be sought in the *Arabidopsis* genome sequence.

Experimental approaches aimed at elucidating the protein content of a cellular compartment require its isolation. Compared to other plant compartments, chloroplasts and mitochondria were relatively easy to obtain in fractions of good purity and detailed subcellular proteomes were obtained from these organelles soon after annotation and publication of the *Arabidopsis* genome. To date, around 800 proteins have been reported from proteome studies to reside in mitochondria (source: SUBA3^1^; Tanz et al., 2013). Similarly, more than 2100 plastid proteins have been reported (source: SUBA3, queried on November 23, 2012). After the initial cataloging of inventory, recent years have seen mitochondria and chloroplasts being subjected to increasingly detailed functional proteomics with the emphasis shifting from discovery to dynamics (reviewed in Braun and Eubel, 2012). Although the predicted peroxisomal proteome of up to 670 proteins (see below) is clearly simpler than that of chloroplasts (>6000 predicted by ChloroP 1.1; Emanuelsson et al., 1999) or mitochondria (>4000 by TargetP 1.1; Emanuelsson et al., 2000), the difficulties involved in obtaining a pure fraction have severely limited the progress in identifying its true components by MS. It was not until 2007 with significant refinements in peroxisome fractionation techniques and the resultant improvement in the quality of the fractions that large-scale proteomics experiments involving peroxisomes became possible (Reumann et al., 2007, 2009; Eubel et al., 2008).

In this review, we will document peroxisome proteome methodologies before arguing that the basic inventory of the peroxisomal proteome is now reasonably well covered, allowing the move toward quantitative and functional studies.

## PROTEOMIC STUDIES OF THE *Arabidopsis* PEROXISOME

### APPROACHES

**FIGURE 2:**
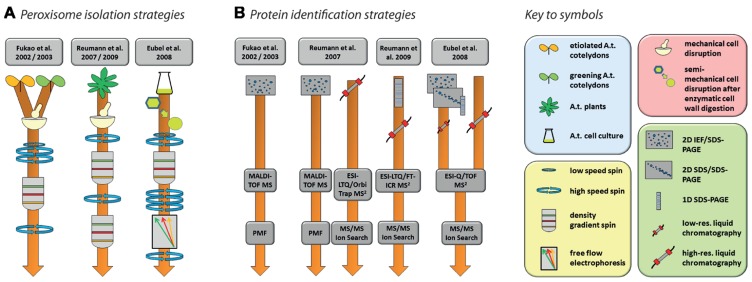
**Strategies for the purification of *Arabidopsis* peroxisomes (A) and the identification of proteins (B) as performed by Fukao et al. (2002, 2003), Reumann et al. (2007, 2009), and Eubel et al. (2008)**. Please refer to the original publication for more detailed information on materials and methods.

In total, five studies have been published with the specified aim of identifying peroxisomal proteins of *Arabidopsis* (Fukao et al., 2002, 2003; Reumann et al., 2007, 2009; Eubel et al., 2008). The experimental strategies involved in the isolation of organelles and identification of proteins are summarized in **Figure 2**. Together, these efforts produced a non-redundant list of 204 proteins (source: SUBA, November 23, 2012) but many more are predicted to be located in peroxisomes (Reumann et al., 2004; Reumann, 2011). Conversely, it is likely that at least some of those that have been identified are contaminants.

Nishimura and colleagues’ pioneering studies in *Arabidopsis* proteomics (Fukao et al., 2002, 2003) reflect the state-of-the-art of proteomics at the beginning of twenty-first century. Using peptide mass fingerprinting (PMF) of proteins separated by two-dimensional iso-electric focusing/sodium dodecyl sulfate polyacrylamide gel electrophoresis (2D IEF/SDS-PAGE), 29 proteins were identified from leaf peroxisomes of greening *Arabidopsis* cotyledons (Fukao et al., 2002), while 19 proteins were found in glyoxysomes of etiolated cotyledons (Fukao et al., 2003). The latter study identified glyoxysomal protein kinase 1 (GPK1), a peroxisome-localized protein kinase. The authors were able to show that a number of glyoxysomal proteins are potentially regulated by phosphorylation events (see Section “Protein Modifications” for further details). Interestingly, the overlap between the two studies consisted of only three proteins, suggesting considerable differences in the protein content of *Arabidopsis* leaf peroxisomes and glyoxysomes. In total, these two studies identified less than 50 proteins. This number includes contaminants as assessed by the combination of high abundances of these proteins in other organelles, the absence of peroxisome targeting signals (PTS; see below), and no obvious relation to expected metabolic activities in peroxisomes.

Since the use of 2D IEF/SDS-PAGE for protein separation actively selects against the identification of membrane proteins for technical reasons, subsequent studies also employed alternative approaches capable of identifying membrane proteins. Reumann et al. (2007) used 2D IEF/SDS-PAGE complimented by shotgun MS to increase the analytical depth of their study of *Arabidopsis* rosette leaf peroxisomes. In addition, they also established a new purification method employing two successive gradients (**Figure 2A**). The first gradient was unusual for organelle separations in that the lysate was spun through a zone of Percoll placed on top of three density layers in which the Percoll concentration was successively reduced toward the bottom while sucrose concentration increased concomitantly. The peroxisomes were retrieved from the bottom of this gradient and then further purified through a second gradient made of seven layers of sucrose solutions with increasing density. The peroxisomes formed a clearly visible white band in the lower part of this gradient. The same isolation procedure on the same tissue was used in a follow-up study (Reumann et al., 2009). In this second study, identification of new proteins was facilitated by first running the peroxisomal proteins in a single lane of a SDS gel, then cutting the lane into 16 slices, each of which was submitted to high-resolution tandem MS (**Figure 2B**). A large proportion of the newly identified putative peroxisomal proteins were then tested for subcellular localization by yellow fluorescent protein fusions (Reumann et al., 2009).

In contrast to the two Reumann studies on *Arabidopsis* leaf peroxisomes, Eubel et al. (2008) approached elucidation of the proteome of plant peroxisomes by choosing non-green *Arabidopsis* cell suspension cultures, thereby eliminating chloroplasts as a major source of contamination in the peroxisomal fraction. With the aim of increasing organelle purity even further, a Percoll gradient was followed by free-flow electrophoresis (FFE; reviewed by Islinger et al., 2010; **Figure 2A**). By using subfractionation of peroxisomes as well as gel (1D and 2D) and non-gel approaches, further novel proteins, including hydrophobic proteins, were discovered by tandem MS (**Figure 2B**).

### CHALLENGES AND PITFALLS

*Arabidopsis* has been the preferred option for subcellular plant proteomics. Isolates of moderate to good quality can readily be obtained for plastids, mitochondria, plasma membrane, and other organelles and compartments from green tissue or non-green suspension cell cultures. Unfortunately isolating good peroxisomal fractions is notoriously difficult in *Arabidopsis*. The reasons for this are likely to be due to some or all of the following: (1) the high content of secondary metabolites found in Brassicaceae potentially interferes with isolation (Kaur and Hu, 2011; Reumann, 2011); (2) peroxisomes are considered to be present in much lower numbers in cells than, for example, mitochondria or chloroplasts (e.g., see Germain et al., 2001; Palma et al., 2009); (3) peroxisomes are thought to be particularly fragile *in vitro* (Palma et al., 2009; Reumann, 2011); (4) losses can be expected to occur due to stresses imposed on the organelles during the isolation procedure; and (5) peroxisomes often physically interact with other organelles (e.g., chloroplasts; Reumann, 2011) and, because of their fragility, using enough force to break these associations may lead to additional peroxisome damage. As such, highly specialized methods were developed for the isolation of organelles from this species that require sophisticated equipment (e.g., FFE) and/or highly optimized procedures as well as detailed knowledge about the stumbling blocks associated with them. Low yields are typical for peroxisome preparations from *Arabidopsis*; this further reduces the error margins for successful preparation of peroxisomes and compromises the purity of peroxisome fractions.

The advantages inherent to *Arabidopsis* (genetic resources, short generation time, easy cultivation, research history) at least partly outweigh the difficulties associated with isolating its peroxisomes and persistence has eventually resulted in acceptable proteomic outcomes. We suggest that a big step forward for peroxisomal proteomics would be if a more generally accessible and well-described isolation technique for *Arabidopsis* were available. Also, we envisage that species such as spinach, pumpkin, soy, and castor oil bean that were all used as early plant models for peroxisome studies will again rise to be of prominence in peroxisome proteomic studies due the increasing availability of sequenced, annotated genomes and their amenability to be used as model species.

## THE *Arabidopsis* PEROXISOMAL PROTEOME

The Arabidopsis 2010 peroxisome project^2^ compiled a “parts list” of 133 confirmed *Arabidopsis* peroxisomal proteins, most of them validated by localization of fluorescent reporter fusion proteins. Although curation of this list has ended, an updated version comparing the *Arabidopsis* proteome with that of rice has recently been published (Kaur and Hu, 2011). This list^3^ includes 163 proteins that fulfill at least two of the criteria of (a) having been identified by MS, and having had their peroxisomal location supported by either (b) the presence of a PTS or (c) localization of fluorescent reporter fusion proteins. As might be expected, this list contains proteins involved in β-oxidation of fatty acids, auxiliary β-oxidation pathways, photorespiration, ROS detoxification, the glyoxylate cycle, branched chain amino acid metabolism, and peroxisome proliferation. It also contains a considerable number of “less traditional” peroxisomal proteins and others with unknown functions, but surprisingly few matrix protein import components are represented. These groups of proteins are summarized in **Figure 1** and will be discussed below in terms of their proteomic coverage and the scope to improve this coverage.

It is particularly noteworthy that the main peroxisomal pathways of *Arabidopsis* (**Figure 1**), as well as almost all of the secondary pathways represented by these 163 proteins, are also among the putative rice peroxisome proteins, at least as evidenced by the presence of rice homologs possessing peroxisome targeting signals (Kaur and Hu, 2011). Such conservation between plant genomes as diverged as rice and *Arabidopsis* suggests that this set of proteins can be taken to comprise the core plant peroxisomal proteome. Indeed it is fair to say that (occasional future surprises notwithstanding) the basic function and proteome of plant peroxisomes is now well established and that this fundamental knowledge will foster more advanced proteomic studies in the future.

### PEROXISOME EVOLUTION, BIOGENESIS, AND PROTEIN IMPORT

Debate on the origin of peroxisomes has centered on two competing hypotheses. Thus, peroxisomes could either have originated as discrete (single-) membrane-bound structures in primitive eukaryotes or alternatively as an engulfed endosymbiont similar to nascent mitochondria and plastids (de Duve, 1969b). The evolution of peroxisomes remained a hotly debated field well into the 2000s. It was only the recent discovery in baker’s yeast (*Saccharomyces cerevisiae*) of *de novo* peroxisome biogenesis from endoplasmic reticulum (ER) that provided strong evidence against an endosymbiont hypothesis for their origin (Hoepfner et al., 2005). As well as budding directly from ER (van der Zand et al., 2012), peroxisomes can proliferate by division of existing organelles and they frequently receive vesicles from the ER that add to the peroxisomal membrane and carry proteins destined for the peroxisomes. The current model of peroxisome biogenesis in plants considers both options of organelle genesis and, accordingly, is referred to as the “ER semi-autonomous peroxisome maturation and replication model” (Mullen and Trelease, 2006). According to this model, in addition to the direct import of proteins synthesized in the cytosol, proteins can also be transported to peroxisomes directly from the ER via the proposed ER/peroxisome intermediate compartments (ERPICs). Understanding of the basics of protein import machinery in plants followed discoveries in mammalian and yeast systems and was largely dictated by the availability of genome sequences that facilitated gene mining for homologs from these systems (Baker and Sparkes, 2005). Genes encoding the essential import and biogenesis machinery are largely conserved across kingdoms indicating that these processes are ancient evolutionary innovations. Peroxisome *de novo* biogenesis involves peroxins PEX3, PEX16, and PEX19 that are all at some time associated with the peroxisomal membrane. Division in *Arabidopsis* involves five different isoforms of PEX11(a–e), at least two fertilization-independent seed (FIS) proteins and three dynamin-related proteins (DRPs) which again are at some time peroxisome-localized (Lingard and Trelease, 2006; Lingard et al., 2008; Hu et al., 2012).

The majority of peroxisome matrix proteins are imported directly from the cytosol by import machinery that recognizes PTS on the proteins. Two types of PTS peptides are responsible for protein targeting to peroxisomes. About 75% of peroxisome-targeted proteins have a so-called PTS1: a C-terminal tripeptide comprised of a non-polar residue in position-1, a basic amino acid in position-2 and a small and uncharged amino acid in position-3 (Reumann, 2004; Lingner et al., 2011). Serine–lysine–leucine (SKL) is the canonical PTS1 but many possible amino acid combinations can function as a PTS1. Improvements in proteomic techniques (e.g., by increased sensitivity of mass spectrometers which produce higher proteome coverage) and confirmation of targeting by other methods (such as targeting of fluorescent fusion proteins) have led to considerable refinement of the definition of the plant PTS1, recognition of the importance of the sequence context of the tripeptide and expansion in the range of permissible PTS1 sequences (Reumann et al., 2012). The second type of peroxisome targeting signal, PTS2, is a nonapeptide usually located within the 20–30 N-terminal residues of proteins that utilize this signal (Reumann, 2004). The consensus PTS2 sequence consists of two pairs of conserved amino acids separated by five non-conserved amino acids and in plants is R[ILQ]x5HL (Kato et al., 1998). As is the case for PTS1s, more recent studies have refined and expanded the range of import-capable PTS2 peptides (e.g., Simkin et al., 2011). PTS2 sequences may also in rare instances be found in the body of the protein (Reumann et al., 2007; Lamberto et al., 2010) and include alternative residues at the second and ninth positions (Kaur and Hu, 2011).

PTS1 tripeptides are recognized by the PEX5 protein that docks with its cargo and is then imported by membrane-bound assembly of PEX13 and PEX14, which form the core import channel. PTS2 import utilizes PEX7 that must first interact with PEX5 before import via the same channels (reviewed in Hu et al., 2012). In both cases, the PEX5/PEX7-cargo complex is imported as a whole into the peroxisomal matrix, with PEX5/PEX7 recycled to the cytosol by a mechanism involving ubiquitination of PEX5 and that utilizes membrane-bound proteins PEX22, PEX2, PEX10, PEX12, and APEM9 as well as other PEX proteins (PEX4, PEX6, and PEX1) that are tethered on the cytosolic side to various protein components of the receptor recycling machinery. After import into peroxisomes, PTS2 sequences are cleaved by the trypsin-like DEG15 protease (Schuhmann et al., 2008); such modifications do not occur with PTS1 signals.

Proteomic analysis by MS has to date been almost singularly unsuccessful at detecting peroxisome-localized components of biogenesis, division and import pathways in *Arabidopsis*. Of the at least 12 protein import-related proteins, only PEX14 was identified in recent *Arabidopsis* studies (Reumann et al., 2007, 2009; Eubel et al., 2008). Similarly, with the exception of PEX11 isoforms (see Reumann et al., 2007, 2009; Eubel et al., 2008), peroxisome biogenesis and division related proteins have not been found by MS-proteomics. Possible reasons for the lack of success in isolating most of the membrane-bound proteins involved in peroxisome biogenesis and protein import in plants are their very low abundances, life stage specific expression, transient associations with peroxisomes, difficulties in the isolation and analysis of peroxisome membranes, and selection against the identification of membrane proteins due to technical limitations (e.g., the use of 2D IEF/SDS-PAGE).

### PROTEINS OF THE CORE PEROXISOME METABOLISM

Peroxisome metabolism is important to all stages of plant growth, including seed development, germination, general growth, and senescence. Peroxisomes can be seen essentially as organelles that sequester reactions producing reactive oxygen species and facilitate benign detoxification of ROS. Other reactions that occur in peroxisomes have almost always been shown to comprise a “service-industry” that recycles reaction intermediates and co-factors. Thus, peroxisomes are primarily β-oxidation (ACX) and photorespiration (glycolate oxidase) machines (**Figure 1**) and the metabolism from these pathways is highly integrated internally and with the rest of the cell. Peroxisomes also house a number of other oxidases that catalyze reactions that are less obviously integrated with “the model peroxisome” metabolism (e.g., sarcosine oxidase, sulfite oxidase, polyamine oxidase, copper amine oxidase, hydroxy acid oxidase and uricase (urate oxidase) (**Figure 1**).

 β-Oxidation provides the primary metabolism remobilizing fatty acids from the oil bodies of oil seed species (such as *Arabidopsis*) to supply energy to seedlings in the initial phases of growth before the onset of photosynthetic energy supply. The sucrose dependence of many β-oxidation pathway mutants is testimony to this (Graham, 2008). The pathway is also responsible for the recycling of fatty acids in senescent plant tissue (Kunz et al., 2009). Peroxisomal β-oxidation is also employed in the modification of other compounds, phytohormones in particular. *De novo* jasmonic acid (JA) synthesis must pass through peroxisomes and precursors of this lipid-derived hormone undergo three cycles of β-oxidation before being exported to the cytosol for conversion to more active forms (Baker et al., 2006). Similarly, indole-3-butyric acid (IBA), regarded as a storage form of auxin, passes through a single round of β-oxidation to form the bioactive indole-3-acetic acid (IAA; Baker et al., 2006). There are numerous peroxisome mutants that display resistance to pro-auxins as a consequence of interruption to the pathway (e.g., Wiszniewski et al., 2009; Strader et al., 2011). Remobilizing IBA to IAA provides at various stages of plant development for a rapid or controlled increase in the pool of bioactive IAA, for example, in germinating seedlings (Strader et al., 2011). Finally, previous suggestions (Boatright et al., 2004; Baker et al., 2006; Kliebenstein et al., 2007) of a peroxisomal contribution to benzoic acid (BA; and as a consequence salicylic acid, SA) synthesis have found new support in the observation of reduced accumulation of BA and BA-CoA in knockouts or ribonucleic acid interference (RNAi) lines of peroxisome-targeted β-oxidation enzymes (Klempien et al., 2012; Lee et al., 2012; Qualley et al., 2012). Given this emphasis on β-oxidation for the organelle, and the increasing diversity of molecules that are altered by the pathway, it is perhaps not surprising that of the total peroxisomal proteome, at least 25% of the predicted (Pracharoenwattana and Smith, 2008) and confirmed (Kaur and Hu, 2011) peroxisomal proteins are directly involved in β-oxidation. This includes glyoxylate cycle enzymes [isocitrate lyase (ICL) and malate synthase], core β-oxidation activities such as acyl-activating enzymes (AAE), ACX, MFP, 3-ketoacyl-CoA thiolase (KAT), and the many subsidiary enzymes in these multigene families such as single-function hydratases, epimerases, and short chain dehydrogenases. This figure of 25% does not include enzymes outside the core pathway such as those of ROS detoxification, malate dehydrogenase (MDH), peroxisomal adenine nucleotide carrier (ANT), enzymes of CoA metabolism, and others that are necessary to sustain β-oxidation and that collectively would clearly significantly increase this proportion.

Once seedlings are established, the primary role of plant peroxisomes is their participation in the photorespiratory pathway. This process serves the detoxification of 2-phosphoglycolate produced by the oxygenase reaction of RubisCO and the salvage of the bulk of its carbon atoms. While the photorespiration pathway is spatially split between chloroplasts, mitochondria, and peroxisomes, the majority of the enzymes directly involved in the recovery of phosphoglycolate (after removal of the Pi group in the chloroplast) are peroxisomal. Six enzymes of photorespiration (including catalase) are located in the peroxisome and at least six different substrates have to be transported across the peroxisome membrane for photorespiration to function properly. This renders peroxisomes the central hub in a biochemical pathway that is so important for the central carbon metabolism of plants that, in terms of carbon flux, it is surpassed only by photosynthesis (Bauwe et al., 2010) and it is no surprise that these proteins are prominently represented in proteomic studies. Despite substantial efforts to reduce the ostensibly wasteful process of photorespiration (e.g., Kebeish et al., 2007; Peterhansel and Maurino, 2011), it is becoming increasingly clear that it is a necessary prerequisite for photosynthesis in C3 plants, that limiting photorespiration inevitably limits photosynthesis itself (Heineke et al., 2001) and that increasing the abundance of photorespiratory enzymes leads to higher rates of photosynthetic carbon fixation and accelerated plant growth (Timm et al., 2012).

### PROTEINS OF OTHER PEROXISOME METABOLIC PATHWAYS

While peroxisomes carry out many hazardous reactions (usually by the actions of ROS-producing oxidases and, to a far lower extent, by reactive nitrogen species (RNS; reviewed in Palma et al., 2009) that need to be shielded away from the rest of the cell, newer data indicate that a range of non-hazardous reactions and pathways also operate in peroxisomes. Potential peroxisomal functions such as protection from herbivore and pathogen attack (Reumann, 2011) are subject of ongoing research.

Aside from ROS-evolving reactions/pathways, peroxisomes have been implicated in co-factor metabolism (CoA), methylglyoxal detoxification, pseudouridine catabolism, and phylloquinone biosynthesis (reviewed in Reumann, 2011). Also, nicotinamide adenine dinucleotide phosphate (NADPH) recycling [including by IDHP (NADP-dependent isocitrate dehydrogenase), oxidative pentose phosphate pathway, the glutathione cycle and NADPH synthesis by NADK3 reviewed in Kaur and Hu, 2011], the initial step of biotin synthesis (Kaur and Hu, 2011) and the mevalonic acid (MVA) pathway of isoprenoid synthesis (Sapir-Mir et al., 2008; Simkin et al., 2011) may take place in peroxisomes (**Figure 1**). Peroxisomal localization of proteins in these pathways has been predicted by informatics and in many cases their targeting potentials were confirmed by green fluorescent protein (GFP) studies. Moreover, with the exception of the MVA pathway enzymes, many of them have also been resolved in proteomic studies (Kaur and Hu, 2011). Since for these non-hazardous pathways distribution to different compartments does not make as much sense as for the ROS-producing processes, one could speculate that the bulk of the reactions carried out in these pathways might also be confined to peroxisomes. At least those proteins that have so far not been positively assigned to any other organelle can be regarded as potential candidates for peroxisome localizations.

Exactly how these processes contribute to peroxisome function and metabolism, or for that matter why they are localized to peroxisomes remains an open question. For example, two enzymes of pseudouridine catabolism (pxPfkB/At1g49350 and IndA/At1g50510) have been found in two different proteome studies, and have been confirmed to have functional PTS peptides (see Reumann, 2011). However, there is no obvious reason why such metabolism should occur in peroxisomes. The substrates and products of the reactions would require transport into the organelle and they do not play any other role in known peroxisomal metabolism. Reumann (2011) suggested that the peroxisome might provide a venue for RNA catabolism away from the actual function and synthesis of RNA. It seems likely that until peroxisome metabolism is better understood, this kind of speculation provides a working model for peroxisomal localization for some of the “non-toxic” pathways.

### PROTEIN MODIFICATIONS

Peroxisomes also modify imported proteins. Such modifications include DEG15 dependent cleavage of PTS2 peptides (Helm et al., 2007; Schuhmann et al., 2008), phosphorylation via the GPK1 kinase (Fukao et al., 2003) and degradation of enzymes such as malate synthase and ICL of the glyoxylate cycle during the transition of peroxisomes from fatty acid degrading to photorespiratory organelles (Lingard et al., 2009). Moreover, the *Arabidopsis* LON2 protease appears to be involved in maintaining matrix protein import by a yet to be determined mechanism (Lingard and Bartel, 2009). It would be particularly interesting to determine the phosphoproteome of peroxisomes and then follow the regulation of phosphatase/kinase activities in the peroxisome. The aforementioned GPK1 and the prediction and observation of a number of other proteases and kinases localized to peroxisomes (Kaur and Hu, 2011) suggest dynamic phosphorylation and dephosphorylation of peroxisomal proteins. At the time of writing, 12 confirmed peroxisomal proteins (At1g04710, At1g06290, At1g07180, At1g20480, At1g20620, At1g20630, At1g23310, At1g49350, At1g54340, At1g65520, At2g06050, and At1g49670) are listed in the PhosPhAt database^4^. Furthermore, PMP38/PXN (AT2G39970, a peroxisome membrane-localized NAD^+^ transporter; Bernhardt et al., 2012), was also found to be phosphorylated (Eubel et al., 2008). We suggest that targeted proteomics could play an important role in analyzing mutants in these protein modification and phosphorylation/kinase genes to determine substrates and extent of processing.

## PEROXISOME PROTEOMICS IN THE FUTURE

With the knowledge gained from previous studies, *Arabidopsis* peroxisome proteomics can be expected to venture in the following directions: (1) search for novel peroxisomal proteins and (2) detailed analyses of peroxisomal proteins in respect to dynamic changes of protein abundances, modifications of peroxisomal proteins, and their potential interactions with other proteins to form temporary or stable protein complexes. Several routes with the potential to further populate the list of plant peroxisomal proteins are discussed and these approaches may be tied to functional proteomic characterization as outlined below.

### IDENTIFICATION OF NOVEL PEROXISOMAL PROTEINS

#### Refinement of peroxisome isolation methodology

In general, allocating proteins to the peroxisomal compartment by experimental data (MS or other methods such as localization of fluorescent protein fusions) provides greater confidence than predictions alone. However, often MS data and even fluorescent reporter proteins produce false-positive results. In the case of MS, the presence of some of the proteins in the data is likely to be due to contamination of the peroxisomal fraction with other organelles. Usually, proteins from contaminating organelles are present in low numbers in the target fraction, but nonetheless they may swamp the analytical sensitivity needed to isolate low abundance proteins of the target organelle. Alternative means are therefore required to isolate organelles to a higher level of purity, both to reduce contamination and to increase the chance of finding rare proteins. One such strategy could be the use of FFE. FFE has been used to further purify mitochondria from yeast and plants, as well as rat peroxisomes (reviewed in Islinger et al., 2010). It has also been successfully employed for the isolation of peroxisomes from *Arabidopsis* cell suspension cultures. Starting from material heavily contaminated with mitochondria, FFE was able to reduce the mitochondrial content by a factor of 5 (based on oxygen electrode assays) while increasing the concentration of peroxisomes threefold (Eubel et al., 2008). Although FFE will face a second major source of contamination in fractions prepared from green leaves (plastids in addition to mitochondria), this approach might also improve the purity of peroxisomes prepared from green plant tissue. In combination with the gradient(s) established for leaf material (Reumann et al., 2007), FFE has the potential to deliver peroxisomes with very low levels of contamination by mitochondria or plastids.

While FFE might be able to reduce contamination, it cannot compensate for the low yields typical of plant peroxisome preparations. The use of more starting material in existing protocols will most probably not produce more isolate since time seems to be an especially critical factor for peroxisome isolation. More starting material means longer preparations and therefore also higher losses. A higher abundance of peroxisomes in the plant material could support higher yields without the need to modify existing procedures. Abiotic stress has been reported to increase the number of peroxisomes per cell (Zhu, 2002) and, while such treatments will inevitably alter the physiology of the plant cell and its organelles, they may represent a viable option to increase yield if the integrity of the organelles is not affected. With these considerations in mind, perhaps the best aim is to counteract peroxisomal breakdown during the isolation procedure. Conditions that stabilize the organelles such as high osmotic strength in the isolation buffers and avoidance of pelleting steps have been reported to be successful (Reumann et al., 2007, 2009). In addition, special care is usually taken to reduce, inhibit, or divert protease activity away from the target proteins in organelle preparations destined for proteomic analyses. However, the same cannot be said for lipid-degrading enzymes set free during cell disruption, which can directly contribute to membrane degradation especially during the first stages of the isolation procedure. This may compromise integrity of the organelles and, finally, the yield of the preparation. Broad-band inhibitors for phospholipases are commercially available but largely omitted in organelle preparations. The use of protease inhibitors in combination with sacrificial phospholipase substrates (such as choline and ethanolamine; Scherer and Morre, 1978) may therefore help to maintain organelle integrity and consequently may also improve peroxisome yield for *Arabidopsis* peroxisome proteomic studies. However, choline and ethanolamine may interfere with subsequent FFE.

#### Bioinformatic approaches to predict novel peroxisomal proteins

Bioinformatics has provided considerable insight into likely upper limits to the size of the *Arabidopsis* peroxisomal proteome. Up to 542 proteins are predicted to contain a PTS1 sequence (SUBA3, November 23, 2012, queried using the PredPlantPTS1 search algorithm of Reumann et al., 2012) and around 110 additional proteins are potentially targeted to peroxisomes by a PTS2, membrane PTS or other means (see Reumann et al., 2004; Kaur and Hu, 2011). The number of potential peroxisomal proteins has expanded considerably in the last 10 years. Reumann et al. (2004) provided the first comprehensive database of putative *Arabidopsis* peroxisome proteins (Araperox^5^), listing 284 proteins based on prediction of PTS1 and PTS2 targeting peptides. Araperox was updated in 2008 to 440 proteins, including another 110 proteins with the newly demonstrated PTS1 signals SSL, SSI, ASL, and AKI (Lisenbee et al., 2005; Reumann et al., 2007), PEX proteins, demonstrated membrane proteins and proteins that are imported using non-standard targeting peptides (e.g., catalase and sarcosine oxidase; Goyer et al., 2004; Oshima et al., 2008). The number of predicted proteins has further increased by refinements to (in particular) PTS1 prediction algorithms (Ma and Reumann, 2008; Lingner et al., 2011; Chowdhary et al., 2012). These studies have taken particular consideration of the context (upstream) of the putative PTS1s and thus identified weak, non-canonical PTS sequences that supported import of proteins into plant peroxisomes. Extensive testing of 23 newly predicted PTS1 motifs suggested unforeseen diversity in plant peroxisome-import competent C-terminal tripeptides (Lingner et al., 2011). The majority of these were tested by fusing enhanced yellow fluorescent protein (EYFP) to the 10 terminal amino acid residues of the predicted proteins or, in a few cases, to the N-terminus of full-length proteins.

While it is possible that the peroxisomal proteome might contain more matrix proteins than are currently predicted, it is equally clear that some predicted peroxisomal proteins are unlikely to localize to the organelle *in vivo*. As a trivial example, the plastid genome encoded rpoC2 (RNA pol) has a strong PTS1 like sequence (SRI) at its C-terminus. In total, 204 proteins have been assigned to the peroxisomal compartment by MS but only 97 of these are found in the list of ~670 proteins predicted (or confirmed by other means) to reside in peroxisomes. This small overlap (<15% of the predicted and <50% of the MS-detected proteins) implies that both the prediction and detection of peroxisomal proteins suffer from false-positive results. It also seems likely that there will be more surprises in the form of unexpected proteins to be assigned to this organelle. Further research and refinement of prediction algorithms (especially for PTS2 sequences) will yield more candidate proteins for the peroxisomal proteome. These bioinformatic works represent major advances in setting the outer boundaries for the plant peroxisomal proteome.

An alternative bioinformatic approach has been to mine the wealth of publicly available transcriptome data for genes that are co-transcribed with genes encoding proteins for core peroxisome functions. This approach was taken in Wiszniewski et al. (2009) to show that numerous previously uncharacterized PTS-encoding β-oxidation genes followed similar patterns of transcriptional expression to other, well-characterized β-oxidation genes. Such data mining for proteins involved in other areas of peroxisome biology could help to provide clues for timing of expression and the function of novel or thus-far undetected putative peroxisomal proteins.

#### Genetic resources

Two classic screens for mutants affected in peroxisome function have been used extensively and both screens can reveal mutants compromised in β-oxidation (Hayashi et al., 1998; Zolman et al., 2000). The first uses the peroxisome-localized conversion of pro-auxins [IBA or 2,4-dichlorobutyric acid (2,4-DB)] into active forms [IAA or 2,4-dichloroacetic acid (2,4-D)]. Mutants in these pathways exhibit root elongation when grown on media containing IBA or 2,4-DB, whereas growth of wild-type roots is inhibited. The second screen utilizes the requirement for β-oxidation to release carbon from fatty acids to fuel germination and seedling establishment. Lesions in this pathway result in seedlings that either do not germinate or fail to establish unless they are grown on media that is supplemented with a sugar carbon source. These early forward genetics studies identified a number of single gene mutants of large effect in these pathways (CTS/PXA1/PED3, KAT2/PED1, etc.). However, many β-oxidation proteins are encoded by gene families that exhibit functional redundancy, and this necessitates targeted (reverse genetic) generation of double- and potentially higher order mutants [e.g., ACX, long chain acyl-CoA synthetase (LACS) families].

Two studies have taken a brute force approach to genetic characterization of putative peroxisome genes. In the first (Wiszniewski et al., 2009), all available mutants for 16 newly predicted (or otherwise uncharacterized) genes with similarity to known β-oxidation genes (as reported in Araperox; Reumann et al., 2004) were screened for response to growth on IBA, 2,4-DB, and sugar-free media. This study yielded new genes in auxin metabolism pathways, but suggested that there were no new single gene knockouts of peroxisome proteins that would display a sugar dependence phenotype. The study also showed that the new auxin pathway genes followed a transcriptional pattern common to many β-oxidation genes. Secondly, Kaur and Hu (2011) hint at a large, unpublished study that took a similar approach with about 50 predicted peroxisome genes and that involved various biochemical, physiological, and cell biological assays aimed at documenting the role of these genes in embryogenesis, peroxisomal protein import, and defense response.

#### Identification of proteins interacting with bona fide peroxisomal proteins

An alternative experimental approach to defining localization is to identify interacting proteins, since these are very likely to be localized in the same compartment. By using an approach not biased by previously defined parameters for peroxisomal targeting, the verification of peroxisome localization of interacting proteins potentially leads to the discovery of so far unknown plant peroxisome proteins and new definitions of functional PTS sequences.

A suite of techniques is available to isolate protein complexes. Antibody- or affinity-tag-based techniques such as co-immunoprecipitation (co-IP) or co-precipitation have been flagged to be of likely utility in expanding proteomic knowledge (Yates, 2000). By these methods, a protein of known localization is used as bait and incubated with protein extracts from fractionated samples to permit interactions and formation of complexes. Subsequently, an antibody against the known peroxisomal protein (e.g., bound to the resin of an affinity chromatography column) is used to pull down the complex, which can be denatured and run on a gel or subject directly to MS detection. An extension of these methods, tandem affinity purification (TAP), has shown particular promise in proteomic applications. TAP involves a two-step affinity purification method that may significantly reduce the incidence of non-specific binding, thus resulting in greater purity of the isolate (Rigaut et al., 1999). The potential for these methods to be used in expanding documentation of the plant peroxisomal proteome is discussed briefly below.

Co-IP has been used in mammalian cells (rat liver) to isolate and confirm interaction of a matrix protein (L-bifunctional enzyme, L-BFE) with catalase (Makkar et al., 2006). Likewise, it was used to demonstrate interactions between a subset of yeast (*S. cerevisiae*) PEX proteins (Eckert and Johnsson, 2003). However, these clearly do not represent high-throughput discovery studies. In a more generic approach, antibodies against a PTS could be used in pull-down experiments. For example, anti-luciferase-PTS1 antibody detects multiple proteins on western blots of purified peroxisome fractions from rat livers (Gould et al., 1990). Using such an antibody in co-IP studies could conceivably yield new proteins, but the antibody is likely to be specific to the particular C-terminal tripeptide (SKL in this case; Gould et al., 1990) and the growing diversity of PTS1 sequences as documented above may limit this approach. Isolation of PTS2-containing proteins would require another suite of antibodies.

TAP has been promoted as a high-throughput method for protein complex discovery. An early application of TAP was a large-scale analysis of protein complexes in yeast (*S. cerevisiae*) in which TAP tags were directly attached to the C-termini of 1739 proteins (Gavin et al., 2002). In total, 589 tagged proteins were purified, 78% of which were associated with potential interaction partners. The experiment involved whole cell extracts, but in principle the interactions occurred *in vivo* and most can therefore be expected to be compartment specific. Thus, proteins and interacting partners were assigned to subcellular compartments, but peroxisomes were not represented amongst them. Likewise, a later study of *S. cerevisiae* purified 2357 tagged proteins and identified over 4000 different proteins involved in interactions, but very few of these were peroxisome-localized (Krogan et al., 2006). These results may reflect (a) that peroxisomal proteins do not form complexes, (b) relatively low abundance of peroxisomal proteins compared to those successfully isolated, or (c) that C-terminal TAP attachment used in both studies masks PTS1 signals with the result that the proteins are not imported and normal complex formation is precluded.

*S. cerevisiae* is well suited to such TAP analysis because homologous recombination can be used to generate large libraries of strains expressing the tagged proteins at approximately native levels (under control of endogenous promoters), and N-terminal tagging could just as well be used to preserve endogenous C-terminal targeting signals. Unfortunately, translating such approaches to *Arabidopsis* and other plants has been problematic, not least because there is no method for homologous recombination of constructs into the genome. TAP tagging in plants thus requires *Agrobacterium*-mediated transformation of individually cloned constructs into wild-type plants, or into mutants in which the native gene has been knocked out to preclude competition of the untagged endogenous protein for binding. Nevertheless, TAP tagging has been promoted for protein complex discovery in plants and the method adapted for plant-specific application (Van Leene et al., 2008). Plant cell cultures have been successfully used with TAP methodology to identify cell cycle component interacting proteins but the process had numerous drawbacks including its complexity, susceptibility to false negatives (due to low abundance, transient expression, or absence of likely interactors in cell cultures) and false positives to highly “sticky” non-specific interactions (Van Leene et al., 2011).

**FIGURE 3:**
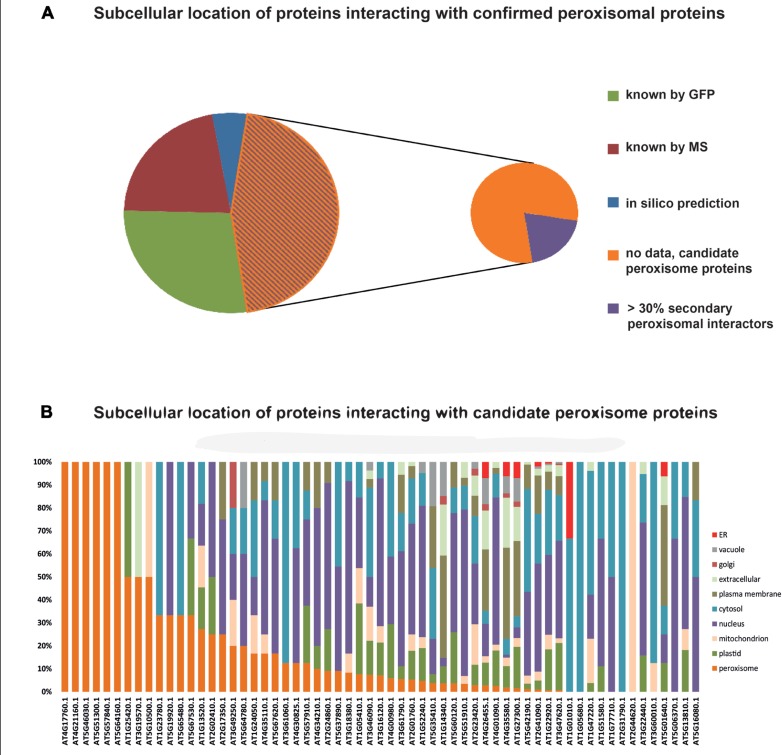
**Subcellular localizations of proteins interacting with confirmed peroxisomal proteins**. One hundred and sixty-three proteins listed by Kaur and Hu (2011) were queried for interacting proteins listed in the SUBA3 database. This returned 133 proteins (**Table 1**) that were then interrogated for their subcellular localization. Thirty-five proteins were allocated to their respective cellular compartment by GFP analyses, 30 by proteomics studies (MS/MS), and eight proteins possessed localization data on the basis of high-confidence predictions. For 60 proteins, no localization was obtainable and therefore they are considered candidate peroxisome proteins **(A)**. In case proteins were assigned to a location by more than one approach, their listing in **Table 1** was done according to the following priorities: GFP > MS/MS > predictions. SUBA was then interrogated with the 60 candidate proteins to determine the localization of their interaction partners, as summarized in the SUBAcon **(B)**. Proteins are sorted by the percentage of peroxisomal interactors, regardless of the overall number of interacting proteins. For six proteins, the bait protein was the only interactor. For some proteins on the list of Kaur and Hu (2011) SUBAcon states a non-peroxisomal localization. Therefore, 13 proteins do not appear to have a single peroxisomal interaction partner although at least the bait protein is expected here.

Data on protein:protein interactions of peroxisomal proteins can also be found in recent global interactome studies. Databases such as the IntAct molecular interaction database (Lee et al., 2010; Kerrien et al., 2012) or the third version of the Arabidopsis Subcellular Database (SUBA3, Tanz et al., 2013), that now incorporates protein interactions, can be queried to detect interaction partners of peroxisomal proteins. Kaur and Hu (2011) have summarized the currently confirmed *Arabidopsis* peroxisomal proteome into a list of 163 proteins (see above). By interrogating SUBA3 (November 23, 2012) with this list, we identified a non-redundant set of 133 proteins with claims for interactions with the original set (**Figure 3A; Table 1**). For 35 of these, the subcellular location was already deduced from fluorescent reporter fusion proteins. Nine of them were exclusively or non-exclusively assigned to peroxisomes. MS assigned 30 other proteins to their respective intracellular locations, and nine of these were also found in peroxisomes. For the remaining 68 proteins, no experimental GFP and/or MS data are stored in SUBA: these proteins are thus candidates for peroxisomal localization. Eight of them were predicted to be peroxisomal by PredPlantPTS1 (Reumann et al., 2012) or Multiloc2 (Blum et al., 2009) and are therefore very likely imported into peroxisomes. On this basis, they should now no longer be considered as candidates but as established peroxisomal proteins (**Table 1**). The remaining 60 proteins were used as queries to interrogate SUBA3 for their interaction partners. Using the putative subcellular localization of the returned interaction partners, as given by the consensus of all localization data for each protein stored in the SUBA3 (SUBAcon), we calculated the percentage of the interacting proteins that are known to be peroxisomal compared to those localizing to other cellular compartments. For 13 candidate proteins, at least a third of the interaction partners consisted of peroxisomal proteins, while this number shrank to nine if a cut-off value of 50% was applied (**Figure 3B; Table 1**). Of these, six proteins had only a single interacting partner and thus by definition had a 100% interaction rate with other peroxisomal proteins: the “bait” was the only confirmed peroxisomal protein. Depending on the level of stringency applied (33, 50, or 100% peroxisomal interactors), these proteins represent the strongest candidates for peroxisomal targeting by this approach and checking their intracellular localization by other means could be a worthwhile undertaking.

**Table 1:** SUBA3 localization data of proteins interacting with confirmed peroxisomal proteins according to Kaur and Hu (2011).

**Interacting proteins without experimental localization data, without predicted peroxisomal localization, and >30% peroxisomal interacting proteins**
AT1G23780.1, AT1G25420.1, AT4G17760.1, AT4G21160.1, AT5G10500.1, AT5G19920.1, AT5G46030.1, AT5G51300.1, AT5G57840.1, AT5G64160.1, AT5G65480.1, AT5G67530.1
**Interacting proteins without experimental localization data, without predicted peroxisomal localization, and <30% peroxisomal interacting proteins**
AT1G01010.1, AT1G05410.1, AT1G05680.1, AT1G13520.1, AT1G14340.1, AT1G22920.1, AT1G24050.1, AT1G27300.1, AT1G47220.1, AT1G51580.1, AT1G52240.1, AT1G77710.1, AT2G01760.1, AT2G02410.1, AT2G17350.1, AT2G23420.1, AT2G24860.1, AT2G31790.1, AT2G41090.1, AT2G44620.1, AT3G16120.1, AT3G18380.1, AT3G22440.1, AT3G46090.1, AT3G47620.1, AT3G49250.1, AT3G60010.1, AT3G61060.1, AT3G61790.1, AT4G00980.1, AT4G01090.1, AT4G26455.1, AT4G30825.1, AT4G34210.1, AT4G35110.1, AT4G35580.1, AT5G01640.1, AT5G06370.1, AT5G13810.1, AT5G16080.1, AT5G35410.1, AT5G37890.1, AT5G42190.1, AT5G51910.1, AT5G57910.1, AT5G60120.1, AT5G64780.1, AT5G67620.1
**Interacting proteins without experimental localization data and with predicted peroxisomal localization**
AT2G01950.1, AT2G33520.1, AT3G03490.1, AT3G58740.1, AT4G14440.1, AT5G56220.1, AT5G65683.1
**Interacting proteins with MS/MS-based localization data**
AT1G03130.1, AT1G06290.1, AT1G12920.1, AT1G20950.1, AT1G43560.1, AT1G68010.1, AT2G13360.1, AT2G26230.1, AT2G35500.1, AT2G42790.1, AT3G12110.1, AT3G14415.1, AT3G14420.1, AT3G21865.1, AT3G26900.1, AT3G58750.1, AT3G60600.1, AT4G02770.1, AT4G22240.1, AT4G28440.1, AT4G35250.1, AT5G11450.1, AT5G25760.1, AT5G38420.1, AT5G46570.1, AT5G55190.1, AT5G56630.1, AT5G65940.1, AT5G66510.1
**Interacting proteins with GFP-based localization data**
AT1G02140.1, AT1G12520.1, AT1G13030.1, AT1G14830.1, AT1G48320.1, AT1G75950.1, AT1G76150.1, AT1G78300.1, AT2G14120.1, AT2G26350.1, AT2G26800.1, AT2G42490.1, AT3G01910.1, AT3G02150.1, AT3G04460.1, AT3G06720.1, AT3G07560.1, AT3G16310.1, AT3G18780.1, AT3G19570.1, AT3G21720.1, AT3G50070.1, AT3G56900.1, AT4G02150.1, AT4G22220.1, AT4G26450.1, AT4G33650.1, AT5G22290.1, AT5G25440.1, AT5G27600.1, AT5G27620.1, AT5G42980.1, AT5G44560.1, AT5G48230.1, AT5G56290.1, AT5G58220.1, AT5G63610.1

### FUNCTIONAL CHARACTERIZATION OF THE PEROXISOMAL PROTEOME

There is more to proteomics than a mere stocktake of the protein content of an organelle. The studies by Fukao et al. (2002, 2003) have clearly shown that we can expect differences in peroxisomes isolated from cotyledons performing autotrophic and heterotrophic metabolism. Except for these early studies (that have more of a qualitative than quantitative character), comparative studies of the plant peroxisomal proteome are, to our knowledge, non-existent. Clearly, obtaining results on changing protein abundances would have a strong impact on our current view of peroxisomes as cellular organelles and their reactions to changing physiological conditions. MS-based comparative studies are traditionally performed by using quantitative approaches such as isobaric labeling [isobaric tags for relative and absolute quantitation (iTRAQ), tandem mass tag (TMT)], heavy mass tags (isotope coded affinity tags, ICAT) and, to a lesser degree, stable isotope labeling (stable isotope labeling by amino acids in cell culture, SILAC). These techniques are quite sensitive and allow quantitation of several thousand proteins at a time. However, due to the relatively low number of peroxisomes within cells and the resulting low average abundance of peroxisomal proteins in cell lysates, coverage of peroxisomal proteins present only in low copy numbers still presents a challenge to quantitative shotgun proteomics. Therefore, isolating peroxisomes from two or more plant populations is still deemed necessary to achieve a better coverage of peroxisomal proteins in shotgun comparative approaches.

A promising alternative to this classical approach may be the use of targeted quantitation of proteins. Using targeted quantitation, low abundance proteins are detectable in tryptic digests of, for example, leaf homogenates without the requirement of first obtaining peroxisome isolates. Targeted absolute or relative quantitation is most commonly performed by employing selected reaction monitoring (SRM; Gerber et al., 2003; DeSouza et al., 2008; Zhi et al., 2011), a technique that originated from the targeted analysis of small molecules and has also become available for peptide analysis. In SRM, only a few peptides specific for the target protein are considered in the MS analysis, resulting in short duty cycles which save analytical time when compared to data-dependent analyses approaches. Thus the elution peaks of the target peptides can be monitored more closely, resulting in higher accuracy quantitation, especially for low abundance peptides. Modern mass spectrometers and knowledge of the retention times of the selected peptides allow the quantitation of up to several hundred transitions in one event and for this reason the technique is often also referred to as multiple reaction monitoring (MRM). Unfortunately, establishing transitions for MRM is a labor-intensive process. However, once this has been achieved, samples can be analyzed in a high-throughput manner allowing the rapid quantitation of proteins in many complex mixtures. The peroxisomal proteome lends itself well to this kind of analysis because protein diversity is rather low compared to other major organelles of the plant cell. This allows relatively good coverage of the peroxisomal proteome with just a single or very few MRM runs. Especially for peroxisomes, establishing good MRM transitions is a worthwhile target, owing to the trials and tribulations associated with their isolation from plant material.

Apart from the peroxisome-specific reasons to employ MRM for the quantitation of proteins, it might also prove advantageous for other organelles. Isolation of the target compartment of a cell is a process that usually takes several hours, during which time unforeseen changes to metabolites, membranes and also to proteins may occur. Additionally, because centrifugation is often used, organellar subpopulations characterized by extreme densities or sizes might constitute the final isolate, differing somewhat from the situation found *in vivo*. Again, due to their fragility, this might affect peroxisomes more severely than other, more stable compartments. Thus, the development of these newer methods to reduce the isolation time and organellar stress prior to analysis will only serve to enhance the value of and possibilities for dynamic proteome studies.

## CONCLUSION

Peroxisomal proteomics in *Arabidopsis* is seriously hampered by limited access to isolated organelles. Due to the low number of peroxisomes in plant cells, most peroxisomal proteins do not rank among the high-abundant proteins. This prevents good coverage of the peroxisomal proteome in shotgun proteomics. Increasingly good predictions of peroxisomal location by different routes will become available in the future. However, experience gained in the past on false-positive allocation of proteins to this organelle has shown that predictions are only suitable for generating candidates for the peroxisomal proteome and that experimental validation of predicted results is still necessary and will most likely remain so. Most of the experimental evidence will be obtained by fluorescent reporter fusion protein assays, but other approaches also lend themselves for this purpose. Laboratories geared toward proteomic studies could use targeted quantitative analysis of candidate proteins from isolated peroxisomes and fractions purified to lesser degrees of homogeneity in order to show enrichment of candidate proteins in peroxisomes.

In order for peroxisomal proteomics to reach the standard of the studies performed on plastids or mitochondria, technical improvements are of paramount importance. Such improvements will be obtained either in the purity and yield of peroxisomal isolates, or by alternative analytical methods to increase proteome coverage. Moreover, investigation of the dynamic changes occurring in the plant peroxisomal proteome may require a completely different approach. Since MS-technology is developing rapidly and advances in the isolation of peroxisomes are comparatively slow, recent and future developments in MS in combination with alternative strategies for identifying members of the plant peroxisomal proteome will be key to a better understanding of the functions and dynamics of this important compartment of the plant cell.

## Conflict of Interest Statement

The authors declare that the research was conducted in the absence of any commercial or financial relationships that could be construed as a potential conflict of interest.

## Footnotes

1 http://suba.plantenergy.uwa.edu.au/ (queried on November 23, 2012)

2 http://www.peroxisome.msu.edu/

3 http://www.ncbi.nlm.nih.gov/pmc/articles/PMC3355810/table/T1/

4 http://phosphat.mpimp-golm.mpg.de/phosphat.html (queried on March 8, 2013)

5 http://www3.uis.no/araperoxv1/

## Abbreviations

1D: one-dimensional
2D: two-dimensional
2,4-D: 2,4-dichloroacetic acid
2,4-DB: 2,4-dichlorobutyric acid
ACX: acyl-CoA oxidase
BA: benzoic acid
BSA: bovine serum albumin
CDS: coding sequence
co-IP: co-immunoprecipitation
DRP: dynamin-related protein
ER: endoplasmic reticulum
ERPIC: ER/peroxisome intermediate compartment
ESI: electrospray ionization
EST: expressed sequence tag
FFE: free-flow electrophoresis
FIS: fertilization-independent seed
GFP: green fluorescent protein
GPK: glyoxysomal protein kinase
IAA: indole-3-acetic acid
IBA: indole-3-butyric acid
ICAT: isotope coded affinity tags
ICL: isocitrate lyase
IDHP: NADP-dependent isocitrate dehydrogenase
IEF: iso-electric focusing
iTRAQ: isobaric tags for relative and absolute quantitation
LACS: long chain acyl-CoA synthetase
MALDI: matrix-assisted laser desorption/ionization
MFP: multi-functional protein
MRM: multiple reaction monitoring
MS: mass spectrometry
MVA: mevalonic acid
NADK: NAD kinase
PAGE: polyacrylamide gel electrophoresis
PMF: peptide mass fingerprint
PTS: peroxisome targeting signal
RNAi: ribonucleic acid interference
RNS: reactive nitrogen species
ROS: reactive oxygen species
SA: salicylic acid
SDS: sodium dodecyl sulfate
SILAC: stable isotope labeling by amino acids in cell culture
SRM: selected reaction monitoring
TAP: tandem affinity purification
TMT: tandem mass tags
